# Evidence of the role of the cerebellum in cognitive theory of mind using voxel-based lesion mapping

**DOI:** 10.1038/s41598-022-09104-0

**Published:** 2022-03-23

**Authors:** Pierre-Aurélien Beuriat, Shira Cohen-Zimerman, Gretchen N. L. Smith, Frank Krueger, Barry Gordon, Jordan Grafman

**Affiliations:** 1 Cognitive Neuroscience Laboratory, Brain Injury Research, Shirley Ryan AbilityLab, Chicago, IL USA; 2 Feinberg School of Medicine, Northwestern University, Chicago, IL USA; 3 Department of Pediatric Neurosurgery, Hôpital Femme Mère Enfant, Hospices Civils de Lyon, Lyon, France; 4 Rockfeller School of Medicine, Claude Bernard University, Lyon, France; 5 School of Systems Biology, George Mason University, Fairfax, VA USA; 6 Department of Psychology, University of Mannheim, Mannheim, Germany; 7 Department of Neurology, Johns Hopkins University School of Medicine, Baltimore, MD USA; 8 Department of Cognitive Science, Johns Hopkins University, Baltimore, MD USA; 9 Departments of Neurology, Psychiatry and Cognitive Neurology and Alzheimer’s Disease, Feinberg School of Medicine, Northwestern University, Chicago, IL USA

**Keywords:** Cognitive neuroscience, Neural circuits, Social behaviour

## Abstract

Theory of Mind (ToM) is a social-cognitive skill that allows the understanding of the intentions, beliefs, and desires of others. There is a distinction between affective and cognitive ToM, with evidence showing that these processes rely on partially distinct neural networks. The role of the cerebellum in social cognition has only been rarely explored. In this study, we tested whether the cerebellum is necessary for cognitive and affective ToM performance. We investigated adults with traumatic brain injury (n = 193) and healthy controls (n = 52) using voxel-based lesion-symptom mapping (VLSM) and by measuring the impact on functional connectivity. First, we observed that damage to the cerebellum affected pure Cognitive ToM processing. Further, we found a lateralization effect for the role of the cerebellum in cognitive ToM with participants with left cerebellar injury performing worse than those with right cerebellar injury. Both VLSM and standard statistical analysis provided evidence that left cerebellar Crus I and lobule VI contributed to ToM processing. Lastly, we found that disconnection of the left thalamic projection and the left fronto-striatal fasciculus was associated with poor cognitive ToM performance. Our study is the first to reveal direct causal neuropsychological evidence for a role of the cerebellum in some but not all types of ToM, processing. It reinforces the idea that social cognition relies on a complex network functionally connected through white matter pathways that include the cerebellum. It supports evidence that the neural networks underpinning the different types of ToM can be differentiated.

## Introduction

Theory of Mind (ToM) is a complex social-cognitive skill. Studies investigating the neural underpinnings of ToM, emphasized the role of cortical regions^1^. ToM abilities seem to mainly rely on the default/mentalizing network. While some studies suggested an overall cortical laterality effect with ToM being linked to the right hemisphere^2^, this is still a matter of debate as a recent meta-analysis by Schurz et al. did not find strong evidence for such a lateralization^3^. A distinction has been made between two different ToM processes: affective ToM (i.e., the ability to infer others’ emotional states and feelings) and cognitive ToM (i.e., the ability to infer others’ beliefs, intentions, and desires), with evidence showing that the two processes rely on partially distinct neural networks^4–6^.

White matter (WM) tracts also have a role in the ToM network. Indeed, maturity of WM connectivity was related to the emergence of mental state attribution in children^7^. Moreover, disconnection in WM pathways appear responsible for impaired ToM performance^8,9^. In patients with autism spectrum disorder (ASD), known to experience impairment in mental state attribution, WM tracts were reported to be affected^10^.

Pioneer work on the role of the cerebellum in social cognition has been done by the Van Overwalle group^11–14^. Still, the role of the cerebellum in social cognition has rarely been directly explored^11^. Buckner et al.^15^ reported that part of the cerebellum was interconnected with the default/mentalizing network that supports ToM in human. Moreover, connectivity between the posterior cerebellum and mentalizing areas has been reported^12,14,16^. Nevertheless, understanding the direct role of the cerebellum in ToM remains challenging. A handful of imaging studies with healthy participants demonstrated cerebellar activation when performing a mentalizing task^17–21^. Yet, only a small portion of the studies reported cerebellar activation during social judgments, including mentalizing^13,22^. Moreover, patient studies demonstrate mixed findings. Some studies show that patients with chronic cerebellar degeneration are impaired on a ToM Task^23,24^. Indeed, it has been shown that inferring the mental state of others through understanding the correct sequences of their actions requires the support of the cerebellum^24^. Others reported impairments but not in every patient^25^. In cerebellar stroke similar discrepancies were reported^26,27^. Moreover, studies focusing on developmental disorders characterized by a mentalizing impairment have reported cerebellar abnormalities and dysfunction of cerebellar-cortical networks^28,29^. Yet, those disorders are complex syndromes and also involve non-cerebellar regions. Finally, there is no clear data on the lateralization of ToM processes in the cerebellum^13,30^.

To our knowledge, studying the role of the cerebellum in several ToM processes (affective-cognitive and purely cognitive) using participants with focal lesions due to Traumatic Brain Injury (TBI) and the Whole-Brain Voxel-based Lesion-Symptom Mapping method has never been done before. Studying participants with a focal TBI evaluated long after the trauma ensures that the primary and long-lasting effect of a lesion to a particular area of the brain can be examined.

In the current study, we tested the role of the cerebellum and of the white matter (WM) tracts that support cortico-cerebellar connectivity in both ToM performance and whether a laterality effect exists for the cerebellum in participants with penetrating TBI (pTBI) in participants from the Vietnam Head Injury Study (VHIS).

To address these questions, we tested a group of individuals with pTBI, as well as healthy controls, on cognitive and affective ToM Tests. Pure Cognitive ToM was measured using the strange stories task, which involve understanding of false beliefs (e.g.: “Simon is a big liar. Simon’s brother Jim knows this; he knows that Simon never tells the truth! Now, yesterday, Simon stole Jim’s ping-pong bat, and Jim knows Simon has hidden it somewhere, though he can’t find it. He is very cross. So he finds Simon and he says, “Where is my ping-pong bat? You must have hidden it either in the cupboard or under your bed, because I’ve looked everywhere else. Where is it, in the cupboard or under your bed?” Simon tells him the bat is under his bed”. Q: Why will Jim look in the cupboard for the bat?”, understanding that if a person never tells the truth, we should not take his advice)^31^, and affective-cognitive ToM was measured using the Faux Pas task, which involve recognizing when someone says something awkward that they shouldn’t have said (e.g. :“Jeanette bought her friend Anne a crystal bowl for a wedding gift. Anne had a big wedding and there were a lot of presents to keep track of. About a year later, Jeanette was over one night at Anne’s for dinner. Jeanette dropped a wine bottle by accident on the crystal bowl, and the bowl shattered. “I’m really sorry, I’ve broken the bowl,” said Jeanette. “Don’t worry, ” said Anne, “I never liked it anyway. Someone gave it to me for my wedding.”, forgetting that a friend gave me a certain bowl as a gift and telling the same friend I don’t like this bowl that someone gave me once)^32^ .

In a recent meta-analysis looking at the neural basis of cognitive and affective ToM^6^, tasks that involved false beliefs (e.g., strange stories task) were categorized as “cognitive”, and tasks that involved observing emotion or pain were categorized as “affective”.

According to the original developers of the task^32^, the faux pas test requires inferring a mental state of belief or knowledge (Someone bought her friend a bowl as a gift) and having *empathic* understanding of how the person in the story would feel (sad that her friend didn’t like the gift that was given to her). While the Faux Pas task does involve cognitive aspects of ToM^5,6,33^, it includes substantial affective components as well, and has been traditionally used as a measure of affective ToM in the literature^32,34–43^. Moreover, A qualitative analysis indicated that the errors made in the faux pas detection task were due to an inability to make emotional representations or impaired ‘affective ToM’ rather than a general ToM impairment^38^. Finally, the inferences one makes regarding others’ mental states include knowledge regarding their thoughts and beliefs, as well as knowledge and empathic understanding of their emotional states and feelings. For example, it may be speculated that while performance of the second-order false belief task requires cognitive understanding of the difference between the speaker's knowledge and that of the listener (knowledge about *beliefs*—“cognitive ToM”), identification of social ‘faux pas’ requires, in addition, an empathic appreciation of the listener's emotional state (knowledge about *emotions*—“affective ToM”)^39^. Therefore, we used it as an affective-cognitive ToM measure, with the affective evaluation being predominant, in the current investigation.

We hypothesized that the cerebellum is part of the network that processes both (affective and cognitive) ToM processes and that disconnection of cerebello-cortical connectivity result in worse ToM performances, independently of left or right cerebellar lesion.

## Methods

### Participants

Participants were drawn from the phase 3 of the VHIS (2003–2008). This longitudinal study followed American combat veterans who suffered brain damage from pTBI in the Vietnam War, as well as neurologically healthy Vietnam combat veterans. Further details regarding the VHIS participants have previously been reported^44^.

Subjects were included in the present study if they completed two ToM tasks: Happe’s Strange Stories Test^31^ and the Faux Pas Stories Test^32^. In total, we collected data from 193 patients with pTBI and 52 control participants. All participants understood the study procedures and gave written informed consent, as approved by the National Institutes of Health Neuroscience Institutional Review Board, Bethesda Naval Hospital and Department of Defense Institutional Review Boards. The Institutional Review Board at Northwestern University approved the current analysis of the data. The data that support the findings of this study are available from the corresponding author upon reasonable request. This study was not preregistered.

### Materials

#### Theory of Mind

While there are many tests for ToM, in this study, we focused on two that are widely used and are considered to the gold standard for assessing affective and cognitive ToM.

##### Happe’s strange stories test

Pure Cognitive Theory of mind was measured using the Happe’s Strange Stories test^31^. The Happe Difference Score was calculated and used as the primary outcome for the Happe’s Strange Stories test. For details, see e-methods.

##### Faux pas stories test

Affective-cognitive ToM was measured using The Faux Pas test^32^. The Faux Pas difference score was calculated and used as the primary outcome for the Faux Pas Stories test. For details, see e-methods.

#### Additional neuropsychological testing

Other neuropsychological tests examined in this study included the Armed Forces Qualification Test (AFQT-7A, 1960). Given that ToM abilities have been shown to covary with working memory^45^ and verbal comprehension abilities^46^, the WAIS Working Memory Index (WMI) and Verbal Comprehension Index (VCI) were used as covariates.

### Neuroimaging assessment and image pre-processing

Neuroimaging assessment and image pre-processing were done using the same method described elsewhere^47^. Detail of the axial computed tomography (CT) acquisition is described in the e-methods. Since metal was retained in the brain due to penetrating wounds or surgical materials, MRI scans could not be acquired. Localization and analyses of the lesion were done as described in the e-methods. Figures were constructed using MRIcroGL v12 (https://www.nitrc.org/projects/mricrogl/).

### Statistical analyses

#### Voxel-based lesion-symptom mapping

A Whole-Brain Voxel-based Lesion-Symptom Mapping (VLSM) analysis was conducted, using the same methods as described in a previous work of our group^1^, on the entire group of pTBI group, in order to test the association between lesion location and ToM performance on the Happe’s Strange Stories test and the Faux Pas Stories test. In VLSM analyses the scores of patients with a lesion in a given voxel is compared to the score of patients without a lesion in this voxel using a t-test. The two primary behavioral outcomes in the VLSM analysis were the Happe Difference Score and the Faux Pas difference score. Additionally, participants’ pre-injury intelligence score, education, WAIS working memory index, WAIS verbal comprehension index and lesion size were used as covariates in order to account for the possible influence of those variables. For detail, see e-methods.

#### Lesion localization and grouping

**Figure 1:**
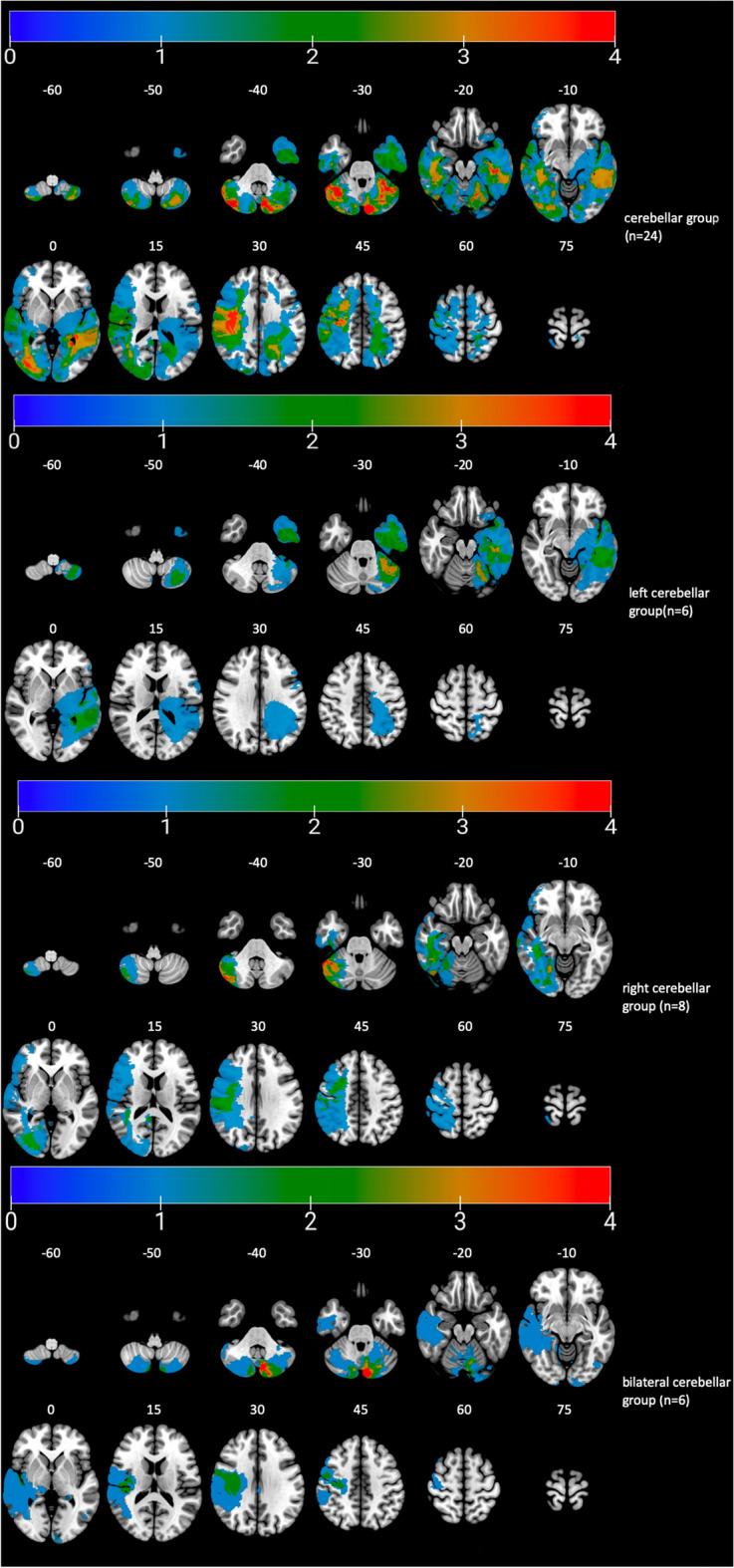
Lesion overlay maps of participant with cerebellar lesion (n = 24) grouped by lesion location. Numbers on the top of the brain slices indicate the z coordinates (MNI) of each axial slice. The color indicates the number of veterans in the group with damage to a given voxel. Images are in radiological space (i.e. right is left). For interpretation of the references to color in this figure legend, the reader is referred to the web version of this article.

**Figure 2:**
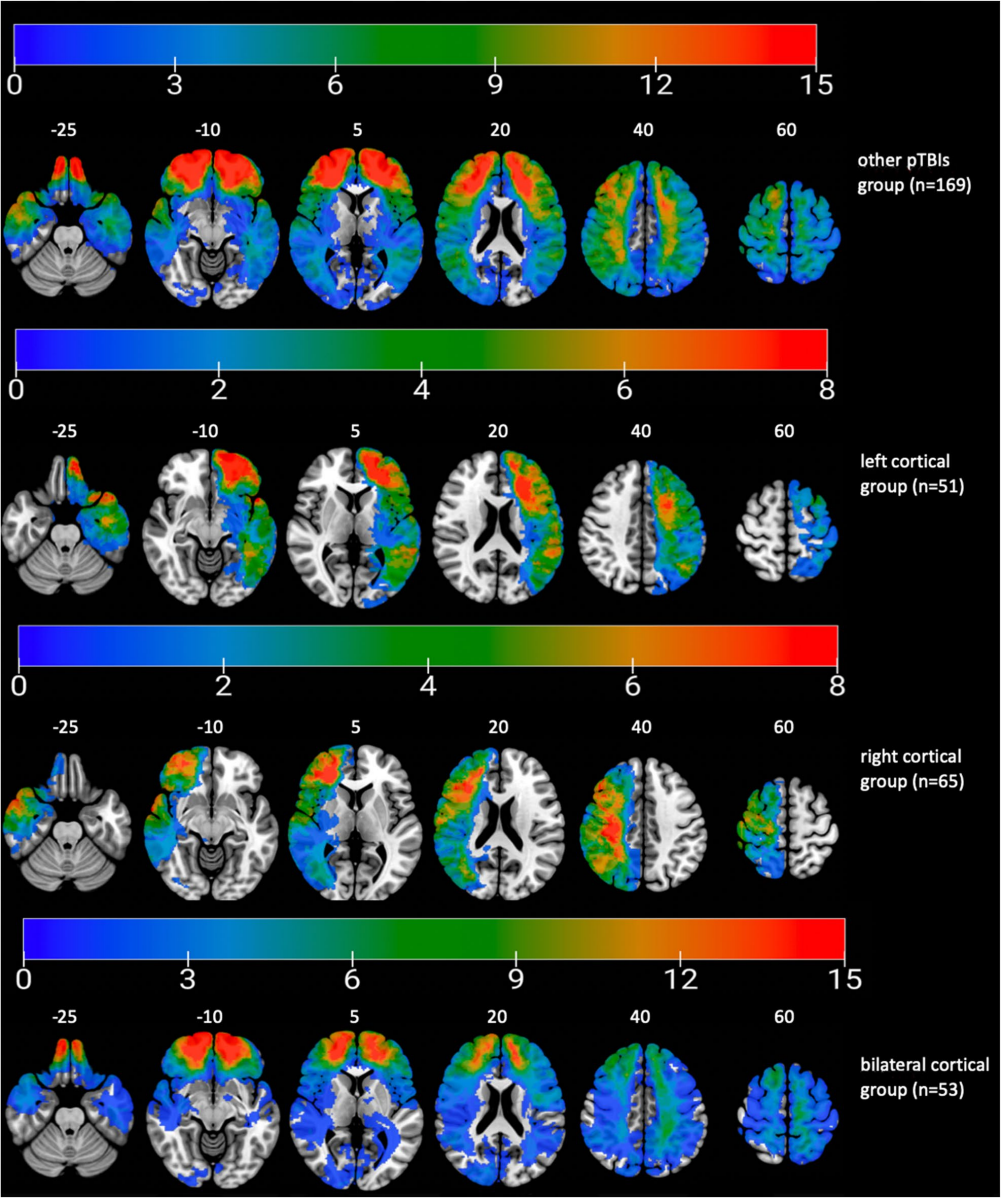
Lesion overlay maps of participant in the other pTBIs group (n = 169) grouped by lesion location. Numbers on the top of the brain slices indicate the z coordinates (MNI) of each axial slice. The color indicates the number of veterans in the group with damage to a given voxel. Images are in radiological space (i.e. right is left). For interpretation of the references to color in this figure legend, the reader is referred to the web version of this article.

We then identified percent volume loss to the cerebellum for each participant in the entire pTBI group (n = 193) using the automated anatomical labeling (AAL). All participants with damage to the cerebellum were selected (Cerebellar Group; n = 24). Note that this cerebellar group included subjects with pTBIs not restricted to the cerebellum (see Fig. 1). Participants with damage primarily to the right cerebellum (r cerebellum; n = 8), left cerebellum (l cerebellum; n = 6) or bilateral cerebellum (b cerebellum; n = 6) were identified (see Fig. 1). Participants with a unilateral cerebellum lesion (right or left) who also had bilateral supra tentorial cortical lesions were excluded from further analysis (n = 4). All of the pTBI participants without a lesion in the cerebellum were selected as a control group (Other pTBIs; n = 169, see Fig. 2). This group was then subdivided into patients with a unilateral left cortical lesion (l Cortical; n = 51), a unilateral right cortical lesion (r Cortical; n = 65) or bilateral cortical lesions (b Cortical; n = 53) for additional analyses (see Fig. 2). To test the lateralization effect on the ToM tasks, both bilateral cortical and cerebellum groups were excluded. Neurologically healthy veterans also served as a comparison group (No Lesion group; n = 52).

#### Behavioral data analysis

Behavioral data analysis was carried out on both cognitive and affective ToM tasks using the same two primary behavioral outcomes of the VLSM analysis namely the Happe Difference Score (to analyze cognitive ToM) and the Faux Pas difference score (to analyze affective-cognitive ToM).

We performed statistical testing using JASP 0.13.1^48^ and significance level was set to *p* < 0.05 (two-tailed unless otherwise specified). Detail of the statistical testing is described in the e-methods.

#### White matter tracts disconnection analysis

To assess the degree to which specific lesions impact brain connectivity we conducted an analysis of WM disconnections contributing to ToM deficits in the cerebellar group. This was done by mapping the normalized lesion from each patient onto tractography reconstructions of WM pathways obtained from a group of healthy controls^49^ and quantifying the probability that the tract was disconnected by a given lesion^50^ using Tractotron software as part of the BCBtoolkit^51^ http://www.toolkit.bcblab.com]. For a similar method see^52^.

## Results

### VLSM

#### Strange stories test

**Figure 3:**
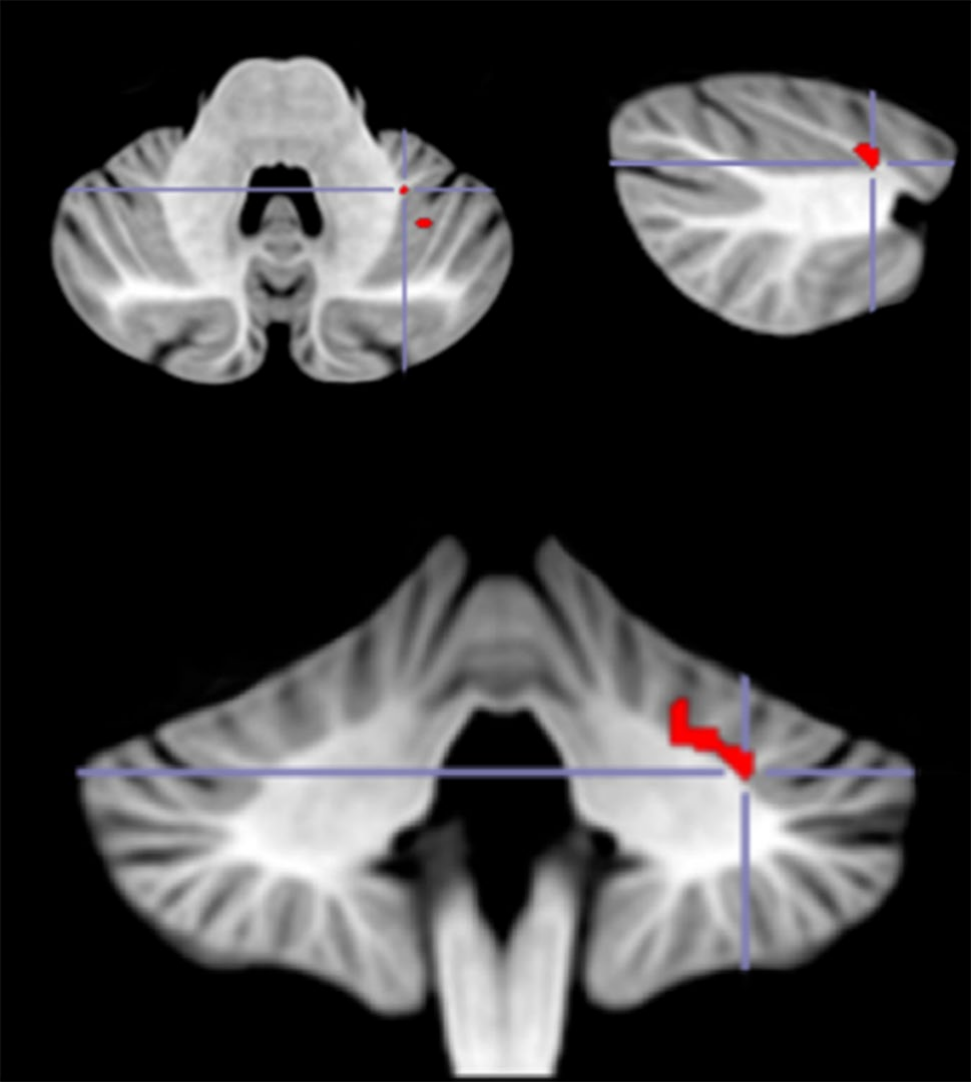
VLSM analysis results. In red are areas of damage in the left lobule VI that were associated with a deficit in cognitive ToM. Peak MNI coordinates for the main cluster (− 30 − 46 − 34).

**Figure 4:**
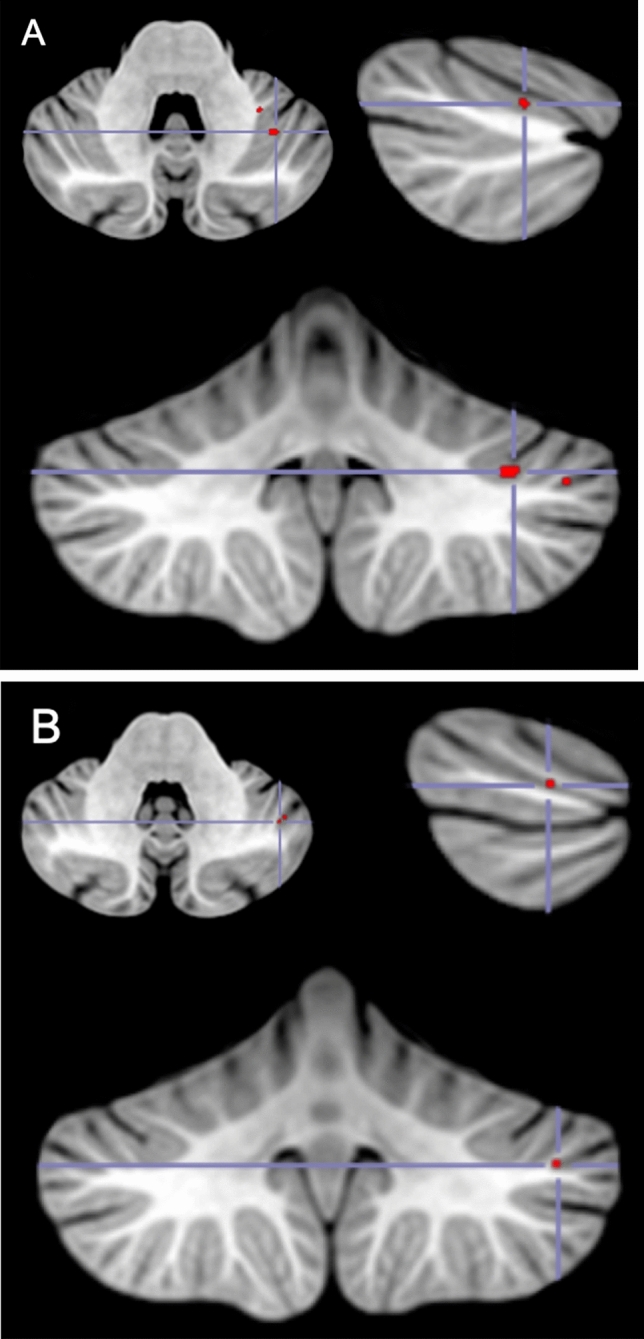
VLSM analysis results. In red are areas of damage in the left Crus I that were associated with a deficit in cognitive ToM. Peak MNI coordinates for the two main cluster (**A** (− 36 − 54 − 34) and **B** (− 44 − 56 − 36)).

A whole-brain VLSM analysis was performed with the Happe Difference Score as the outcome, and the following five measures as covariates: pre-injury intelligence score, WAIS working memory index score, WAIS verbal comprehension index score and lesion size. The VLSM analysis revealed three significant clusters in the cerebellum. The largest cluster (volume = 34 voxels, Max t = 2.07) was located within the left lobule VI. The peak MNI coordinates were (− 30 − 46 − 34), and the center coordinates were (− 25 − 47 − 30, see Fig. 3). The two other clusters were smaller (volume = 2 voxels, Max t = 1.85) and located within the left Crus I. The peak MNI coordinates were (− 36 − 54 − 34 and − 44 − 56 − 36 respectively), and the center coordinates were (− 35 − 54 − 34 and − 45 − 55 − 36 respectively; see Fig. 4a,b).

#### Faux pas stories test

A whole-brain VLSM analysis was performed with the Faux Pas difference score as the outcome, and the following five measures as covariates: pre-injury intelligence score, WAIS working memory index score, WAIS verbal comprehension index score and lesion size. This VLSM analysis revealed no significant clusters in the cerebellum.

### Group analysis

#### Demographics and additional neuropsychological tests

Demographics and neuropsychological testing results of veterans with pTBI (n = 193) and the No Lesion group (n = 52) are presented in Table 1. The following ANOVA between the cerebellar group, the other pTBI group and the No Lesion group revealed that the groups differed on the WMI score (F_(2, 239)_ = 6.94, *p* = 0.001 η^2^ = 0.05) with the No Lesion group scoring higher than the other pTBI group (P_bonferroni_ < 0.001) but not the cerebellar group (P_bonferroni_ = 0.08). Nonetheless, all three groups performed within the normal range for this test. In addition, total brain volume loss did not differ among the cerebellar group and the other pTBI group (U = 2354, *p* = 0.18, RBC = 0.17).

**Table 1:** Demographics and neuropsychological measures (mean (SD)) for veterans with penetrating traumatic brain injury (pTBI) and healthy controls.

Variables\group	pTBI n = 193	No lesion group n = 52	Statistics
**Demographics**			
Age (years)	58.23(2.84)	59.13 (3.44)	U = 4526.5, *p* = 0.35, RBC = − 0.09
Education (years)	14.77 (2.54)	15.22 (2.51)	U = 4526.5, *p* = 0.35, RBC = − 0.09
Handedness (R:L:A)^a^	160:27:6	42:7:3	*X*^*2*^_*(2,N*=*193)*_ = 5.899, *p* = 0.21
**Neuropsychological**			
Pre-injury IQ^b^	60.71 (25.44)	68.06 (21.52)	U = 2413.5, *p* = 0.13, RBC = − 0.17
Cognitive Theory of Mind^c^	0.40 (3.29)	− 0.08 (3.45)	t_(243)_ = 0.93, *p* = 0.35, d = .145
Affective Theory of Mind^d^	− 21.95 (15.06)*	− 16.11 (11.70)*	U = 3816.5, *p* = 0.08, RBC = − 0.24
Working memory^e^	97.21 (14.91)*	105.71 (12.57)*	U = 3267, *p* < 0.001, RBC = − 0.33
Verbal comprehension^f^	106.48 (15.64)	110.02 (11.86)	U = 4417, *p* = 0.18, RBC = − 0.12

*Denotes significant group difference *p* < 0.05.

^a^Handedness (L:R:A), Left, right and ambiguous.

^b^Percentile score of Armed Forces Qualification Test (AFQT).

^c^Happe’s Strange stories task: difference score between ToM and Control condition. Lower score reflects lower ToM performance.

^d^Faux Pas stories task: difference score between ToM and Control condition. Lower score reflects lower ToM performance.

^e^WAIS Working Memory Index score.

^f^WAIS Verbal Comprehension Index score.

We next compared 4 participant groups based on lesion location: r cerebellum, l cerebellum, r cortical and l cortical (See Lesion localization and grouping Section for grouping procedure). Demographics and neuropsychological testing results of the group analysis are shown in Table 2. No significant difference on total years of education (F_(3,126)_ = 0.72, *p* = 0.54, η^2^ = 0.02), pre-injury IQ scores (F_(3,117)_ = 0.76, *p* = 0.0.52, η^2^ = 0.02), verbal comprehension score (χ^2^_(3, n=126)_ = 5.83, *p* = 0.12) and working memory score were found (χ^2^_(3, n=125)_ = 6.13, *p* = 0.11). Once again total brain volume loss did not differ across the 4 groups (F_(3,125)_ = 1.14, *p* = 0.34, η^2^ = 0.03).

**Table 2:** Demographics and neuropsychological measures (mean (SD)) for veterans grouped by lesion location.

Variables\group	Right cerebellar N = 8	Left cerebellar N = 6	Right cortex N = 65	Left cortex N = 51
**Demographics**				
Age (years)	58.87 (1.64)	58.83 (0.98)	58.37(2.89)	58.47 (2.72)
Education (years)	15.37 (1.60)	13.50 (1.76)	14.88 (2.57)	15.05 (2.84)
Handedness (R:L:A)^a^	6:2:0	5:1:0	52:10:3	45:5:1
**Neuropsychological**				
Pre-injury IQ^b^	62.12 (82.92)	51.83 (28.37)	61.61 (25.30)	66.35 (23.26)
Cognitive Theory of Mind^c^	2.37 (2.67)*	− 0.50 (3.27)*	0.03 (3.29)	0.88 (3.57)
Affective Theory of Mind^d^	− 19.25 (10.07)	− 23.00 (16.48)	− 19.63 (14.08)	− 24.14 (18.67)
Working memorye	98.00 (12.22)	85.50 (8.48)	100.32 513.33)	96.10 (16.45)
Verbal comprehension^f^	114.88 (8.37)	96.33 (6.83)	108.71 (14.46)	106.29 (19.36)
Total brain volume loss (cc^3^)	45.79 (43.45)	57.62 (79.22)	29.24 (34.02)	31.89 (44.22)

*Denotes significant group difference *p* < 0.05.

^a^Handedness (L:R:A), Left, right and ambiguous.

^b^Percentile score of Armed Forces Qualification Test (AFQT).

^c^Happe’s Strange stories task: difference score between ToM and Control condition. Lower score reflects lower ToM performance.

^d^Faux Pas stories task: difference score between ToM and Control condition. Lower score reflects lower ToM performance.

^e^WAIS Working Memory Index score.

^f^WAIS Verbal Comprehension Index score.

#### Theory of mind tasks

Regarding the ToM tasks, results of veterans with pTBI and the HC were also comparable on their performance in the Happe’s Strange Stories test (t_(243)_ = 0.93, *p* = 0.36, d = 0.15) but not on their performance in the Faux Pas Stories test (U = 3816.5, *p* = 0.008, RBC = − 0.24). The following ANOVA between the cerebellar, the other pTBI and the No Lesion group revealed a difference within the groups on the Faux Pas Stories Test (F_(2, 242)_ = 3.33, *p *= 0.04 η^2^ = 0.03). However, only the difference between the No Lesion group, scoring higher, and the other pTBI group (P_bonferroni_ = 0.03) survived the Bonferroni correction.

In order to confirm the lateralization of the cerebellar involvement in the ToM processes found on the VLSM analysis, we compared the performance on both ToM tasks between participants with left or right cerebellar lesions. A significant difference was found on the cognitive ToM task with participants with a left cerebellar lesion demonstrating a lower score on the Happe’s Strange Stories test (one-tailed t-test; t_(12)_ = 1.81, *p* = 0.05, d = 0.98). The mean Happe Difference score for the l cerebellar group was negative (M = − 0.5, SD = 3.271) reflecting a mean deficit in the task in the l cerebellar group (see above Happe’s strange stories test Section) whereas the mean score for the r cerebellum was positive (M = 2.37, SD = 2.67). Note here that, the mean ToM condition score for the l and r cerebellar group was respectively 7 (SD: 3.2) and 11 (SD: 2.9) (max score 16) and the mean Physical condition score difference for the l and r cerebellar group was 7.5 (SD: 4.0) and 8.6 (SD: 2.4) respectively (max score 16). No norms exist for this task but the mean score of the l cerebellar group was lower than the mean score of the stroke patients (with cognitive ToM deficit) reported by Happe et al. in her original publication (mean score: 10.6, SD: 3.4)^53^. However, no significant difference was found on the Faux Pas Stories test between the groups (t_(12)_ = 0.53, *p* = 0.61, d = 0.29).

Next, we conducted linear regression analyses to test whether damage to left Crus I and lobule VI was specifically associated with cognitive ToM performance. The regression model included the Happe differences score as the dependent variable, and the following as covariates: pre-injury intelligence score, years of formal education, WAIS working memory index score, WAIS verbal comprehension index score, percent damage to left Crus I and lobule VI and the total volume loss. Overall, the model explained a significant proportion of variance in cognitive ToM performance (R^2^ = 0.79, F_(7,14)_ = 3.76, *p* = 0.05), with more damage to the left Crus I predicting lower ToM performance (β = 0.73; t = 3.06, *p* = 0.02,one-tailed) as well as more damage to the left lobule VI (β = − 0.67; t = − 3.32, *p* = 0.01,one-tailed). No other covariate contributed significantly to the model including right cerebellar structures.

The same linear regression analyses were conducted for the performance on the Faux Pas Stories Test. None of the covariates contributed significantly to the model including the right or left cerebellar structures.

### White matter tracts disconnection

Cerebellar group participants’ lesions were compared to an atlas of WM connections in order to identify the probability of tract disconnections^49,51^. The percentage of patients with disconnected tracts was calculated separately only for patients with and without deficits in cognitive ToM (determined based on zero as a cut-off score, difference score equal or higher than 0 reflects no deficit, score < 0 reflects deficit), for patients with a left cerebellar lesion (n = 6) and a right cerebellar lesion (n = 8). Only cognitive ToM was explored as no significant results were found either on the VLSM or on group analysis on the Faux Pas Stories Test. We compared the groups of patients with and without presumed damage for each WM tract separately, using a chi-square test. This analysis revealed that disconnections of the left Thalamic projection (*X*^2^ (1, *N* = 14) = 4.20 *p* = 0.04), and the left Fronto-Striatal fasciculus (*X*^2^ (1, *N* = 14) = 4.20 *p* = 0.04) were modestly associated with a poorer performance in the cognitive ToM task. However, these comparisons did not survive Bonferroni correction for multiple comparisons.

## Discussion

In this study we explored the role of the cerebellum in ToM. We found that a lesion to the left cerebellum, and more specifically to the left Crus I and lobule VI, led to a deficit in cognitive ToM. We also found that disconnection of the cerebello-cortical pathways through the left fronto-striatal tract and the left thalamic projection were more likely to result in cognitive ToM deficits.

Overall, these findings suggest that the left cerebellum contributes to the cognitive processes of mental state attribution.

### The difference between cognitive and affective Theory of Mind

To our knowledge, our study is the first to report that the cerebellum might play a different role in the different networks supporting different ToM processes. This is in line with previous findings suggesting a distinct neural network for each ToM process^54^. Our study adds a novel region to these networks.

### The role of the cerebellum in theory of mind neural network

It is now well established that the cerebellum plays a role in more than motor functions as described by Schmahmann et al. in 1998^55^. In the original description of the Schmahmann syndrome (posterior fossa syndrome), attention was drawn to the executive function impairments even if alteration of affective processes were also involved. The role of the cerebellum in social cognition emerged in the last decade. In a first meta-analysis, Van Overwalle et al. reported that cerebellar “social” clusters overlapped with nonsocial function clusters^13^. Therefore, they argued that the cerebellum provides a domain-general support of social cognition^13^. However, after reporting that their mentalizing clusters were located mostly within the default network^15^*,* they concluded that the cerebellum provides a domain-specific process for social cognition^12^.

Regarding the anatomical localization of cerebellar regions that are associated with mentalizing, we found a ToM cluster only for the cognitive task, and only in the left cerebellar hemisphere. Our clusters were more lateral than previously described. When compared to the ToM activation map of King et al. our clusters are outside the ToM map and are all within region 6 (Active maintenance, divided attention, verbal fluency)^30^. Also, when compared to the Buckner et al. 7 network map, our clusters are not within the default network but within the ventral attention network and the executive/cognitive network^15^. These differences may be related to the difference in the subject sample, the tasks used and our analytic approach. However, even in the previous studies, there were inconsistencies regarding which cerebellar lobules were involved, especially Crus II and if a laterality effect existed. Van Overwalle et al.^13^ reported that person mentalizing clusters were associated with the right Crus I, left and right lobule VI and right lobule IV. But when using another type of analysis, the clusters were encompassed in left and right Crus II^11^. The same group recently reported the role of the Crus II in a meta-analysis on cognitive and affective ToM judgments about other persons and the self^22^ and also on the role of Crus I, in addition to Crus II when predicting social action sequences from trait information^17^. Because the meta-analysis by Van Overwalle et al.^22^ did not find differences in both ToM processes in Crus II leaves open the possibility that these differences can be found elsewhere in the cerebellum as reported in our study. King et al.^30^ reported that most of the ToM activations were within left and right Crus I and II (with a larger lateral spread on the right side) but with extension to right lobule VI and IX and to the midline (vermis). However, in a recent functional neuroimaging study, it was reported that impairment on a complex ToM task was associated with decreased volume of the left lobule VI^56^. Our results corroborate these latter findings.

Thus, multiple cerebellar lobules are involved in the ToM neural network. We did not find clusters in all of the lobules reported previously, which does not imply that these others lobules are not involved in the network.

### The role of the cerebellum in cognitive theory of mind

It is widely accepted that the cerebellum has a role in predicting motor outcome and signaling the cortex when errors are detected, in order to update the motor signal and reduce errors in future movements^57^. Similarly to motor activity, social behavior also require anticipation and adaptation^58,59^. Motor and space processing may help characterize environmental constraints on social behavior by processing of physical boundaries, agent sequences, and coordination. In particular, adaptation and prediction of the behavior of the self and others could be a very specific contribution that the Cerebellum makes to understanding the intent of others^23,60^. This idea supports previous arguments claiming that the cerebellum regulates cortical functions for complex social behaviors by enhancing the feedforward control that is necessary to perform these functions correctly^61,62^. Moreover, it has been also shown that identifying and reconstructing the sequence of social behaviors requires mentalizing which strongly recruited the posterior cerebellum compared to other control conditions without sequencing or without social mentalizing content^17–21^. One hypothesis why the cerebellum may be involved in cognitive but less in affective ToM results follows directly from this interpretation. Cognitive ToM is a more complex form of ToM compared to affective ToM because it subserves higher-order cognitive and metacognitive processes^56^. Affective ToM tasks would require less prediction and therefore, would not tax the cerebellum as much as cognitive ToM. The Clausi et al. findings support this by reporting no impairment in a task that assesses the ability to attribute emotions to others in a social context and argue that it was because the task requires reduced prediction and interplay between cognitive and emotional aspects^63^.

Another hypothesis is that the role of the cerebellum in the cognitive ToM task is in controlling the sensorimotor aspects of ToM. Indeed, it was reported that Cognitive but not affective ToM deficit was also related to working memory performance^64^. Our group has shown that the role of the cerebellum in executive function is *supportive* since it appears to primarily compute the motor component of working memory^47^. Also, in patients with cervical dystonia, it was reported that only cognitive ToM was impaired in those with tremor compared to those without motor impairment^65^. Therefore, one could hypothesize that cognitive ToM tasks rely more upon sensorimotor control than affective ToM tasks.

### The role of the white matter tracts

The cerebellum is interconnected with the cerebrum via cerebello-cortical WM loops. In our study, the WM disconnection analysis revealed that damage to the WM pathways that include the “relay” structures of the cerebello-cortical WM connection, namely the thalamus and the striatum, were associated with poor performance in cognitive ToM. This finding suggests an important role for the cerebellum in the neural network that supports cognitive ToM and that integrity of the cerebello-cortical tracts is essential for cognitive ToM.

### Limitations

All participants were male veterans and mostly Caucasian, therefore this limit our ability to generalize the results to other populations. Moreover, there are documented sex differences in ToM^66^ which could not be addressed. As happens in longitudinal studies, participants in the current phase of the study are likely to have recovered better from their injury than patients who would be assessed shortly after their injury. Yet, our ability to identify impairments in this set of patients suggest that cerebellar damage can lead to poorer ToM ability even after several decades. As noted in the methods section, only CT images were used, but MRI and DTI tractography should also be used for white matter pathways identification in future studies with other patient populations. Finally, we enrolled a small number of participants with pTBI *limited* to the cerebellum which could have precluded obtaining significant results.

## Conclusions

This study is the first to provide direct causal neuropsychological evidence for an important role of the cerebellum in cognitive ToM processing. Our results indicate that human social cognition relies on a complex network functionally connected through WM pathways that include the cerebellum. It provides evidence that it is the left cerebellar Crus I and lobule VI that contributes to cognitive mental attribution. It also supports evidence that the neural networks underpinning cognitive and affective ToM can be differentiated.

## Supplementary Information

Supplementary Information.

## Data Availability

The data that support the findings of this study are available from the corresponding author upon reasonable request. This study was not preregistered.
