# Supplementary material for: Evidence of the role of the cerebellum in cognitive theory of mind using voxel-based lesion mapping
Source: Sci Rep. 2022 Mar 23;12:4999. doi: 10.1038/s41598-022-09104-0 (PMC8943209; doi:10.1038/s41598-022-09104-0)
Supplement: Supplementary file 1 — Supplementary Information. [file 41598_2022_9104_MOESM1_ESM.docx]

**e-methods : Materials**

2.2.1 Theory of mind

2.2.1.1 Happe’s Strange Stories Test

Each participant was presented with 16 stories, eight ToM stories and eight control stories, which were selected from the 24 original stories and had been used in prior imaging and neuropsychological studies (1,2). Both sets of stories involved people and required attention to sentence meaning, memory, and question answering, however only the ToM questions were based on understanding the beliefs and intentions of characters in the stories, while the control story questions were based on physical inferences made about the story. Stories are of comparable difficulty in healthy young adults (3).

The following story represents an example of a selected ToM story (story number 21): “*Simon is a big liar. Simon’s brother Jim knows this; he knows that Simon never tells the truth! Now, yesterday, Simon stole Jim’s ping-pong bat, and Jim knows Simon has hidden it somewhere, though he can’t find it. He is very cross. So, he finds Simon and he says, “Where is my ping-pong bat? You must have hidden it either in the cupboard or under your bed, because I’ve looked everywhere else. Where is it, in the cupboard or under your bed?” Simon tells him the bat is under his bed. Q: Why will Jim look in the cupboard for the bat?*”

The following story represents an example of a selected physical (control) story (story number 12): “*A burglar is about to break into a jewelers' shop. He skillfully picks the lock on the shop door. Carefully he crawls under the electronic detector beam. If he breaks this beam it will set off the alarm. Quietly he opens the door of the storeroom and sees the gems glittering. As he reaches out, however, he steps on something soft. He hears a screech and something small and furry runs out past him, towards the shop door. Immediately the alarm sounds. Q: Why did the alarm go off?”*

All 8 stories of each type were administered as a group, but the order of the two sets was counterbalanced among participants. For each story, participants were instructed to read the story and then answer a question on a separate page. Participants received two points for each fully explicit correct answer, one point for partial, implicitly correct answers, and zero points for an incorrect answer or no response.

Three scores were calculated for each participant: 1) ToM story score (the sum of the scores for each ToM story question, range 0-16); 2) physical story score (the sum of the scores for each physical story question, range 0-16) and 3) the Happe Difference Score, calculated as the difference between score 1 and 2. This score was used as the primary outcome for the Happe’s Strange Stories test, with zero reflecting no difference in performance on the ToM and Physical stories, and a negative score reflecting a lower score on the ToM stories compared to the physical stories condition.

2.2.1.2 Faux Pas Stories Test

The Faux Pas test included 10 stories in which a social “faux pas” occurred (“faux pas” stories) and 10 control stories in which no social “faux pas” occurred (“no-faux pas” stories).

The following story represents an example of a selected Faux Pas story (Story number 14): *“Jeanette bought her friend Anne a crystal bowl for a wedding gift. Anne had a big wedding and there were a lot of presents to keep track of. About a year later, Jeanette was over one night at Anne’s for dinner. Jeanette dropped a wine bottle by accident on the crystal bowl, and the bowl shattered. “I’m really sorry, I’ve broken the bowl,” said Jeanette. “Don’t worry, ” said Anne, “I never liked it anyway. Someone gave it to me for my wedding.”1. Did anyone say something they shouldn't have said or something awkward? If yes, ask: 2.Who said something they shouldn't have said or something awkward? 3.Why shouldn't he/she have said it or why was it awkward? 4.Why do you think he/she said it? 5.Did Anne remember that Jeannette had given her the bowl? 6.How do you think Jeanette felt?”*

The following story represents an example of a selected Control Story without a faux pas (story number 5): “*Bob went to the barber for a haircut. "How would you like it cut?" the barber asked. "I'd like the same style as I have now, only take about an inch off," Bob replied. The barber cut it a little uneven in the front, so he had to cut it shorter to even it out. "I'm afraid it's a bit shorter than you asked for," said the barber. "Oh well," Bob said, "it'll grow out." 1. Did anyone say something they shouldn't have said or something awkward? If yes, ask: 2. Who said something they shouldn't have said or something awkward? 3. Why shouldn't he/she have said it or why was it awkward? 4. Why do you think he/she said it? 5. While he was getting the haircut, did Bob know the barber was cutting it too short? 6. How do you think Bob felt? Control question: 7. In the story, how did Bob want his hair cut? 8. How did the barber cut his hair?”*

To reduce memory demand, participants could read the story while the stories were read to them. When a “faux pas” was identified, five clarifying questions were presented to evaluate the understanding of the mental states and emotions of the agents involved in the stories. Each “faux pas” story question correctly answered was scored as 1, resulting in a maximum score of 6 for each story. The “no-faux pas” stories were given a score of 2 if they were correctly identified as not containing a faux pas. Two more control questions were asked for all 20 stories to confirm that the participant had a factual understanding of the stories.

Three scores were calculated for each participant: 1) the percentage of correct Faux Pas stories; 2) the percentage of correct control question for the Faux Pas stories and 3) the Faux Pas difference score, calculated as the difference between scores 1 and 2. In this score zero reflects no difference in performance on the Faux Pas stories Question and the Faux Pas stories Control Question, and a negative score reflecting a lower score on the Faux Pas stories Question compared to the Faux Pas stories Control Question. This score was used as the primary score for the Faux Pas Stories test.

**e-methods : Neuroimaging assessment and image pre-processing**

Axial computerized tomography (CT) scans without contrast were acquired using a GE Medical Systems Light Speed Plus CT scanner at the Bethesda Naval Hospital. Magnetic resonance imaging (MRI) could not be performed with patients in this study due to the possible presence of metal fragments from shrapnel or other missile fragments, residual metallic surgical clips or cranioplasties from surgery. Images were reconstructed with an in-plane voxel size of 0.4 x 0.4 mm, an overlapping slice thickness of 2.5 mm and a slice interval of 1 mm. We determined lesion location and volume from CT images using the Analysis of Brain Lesion (ABLe) software (4) contained in MEDx v3.44 (Medical Numerics, Germantown, MD) with enhancements to support the Automated Anatomical Labeling (AAL) atlas (5). All areas from the AAL atlas were used to determined lesion location. A trained neuropsychiatrist manually traced individual lesions, which were then reviewed by a researcher who was blind to the results of the Phase 3 evaluation (JG). Scans were spatially normalized to Montreal Neurological Institute MNI space (6) using the Automated Image Registration program (7) using a 12-parameter affine model on de-skulled CT scans. We did not include voxels with lesions in the spatial normalization procedure in order to reduce image distortions. Lesion volume was calculated by summing the traced areas in all relevant slices of the CT image, and then and multiplying by slice thickness.

**e-methods : Statistical analyses**

2.4.1 Voxel-based lesion-symptom mapping

In order to have sufficient statistical power and to be able to test regions all over the brain, voxels that did not contain at least 4 patients with damage were excluded from the analysis. To correct for multiple comparisons, a false discovery rate (FDR) correction of 0.05 was used. FDR is widely used for analysis of functional neuroimaging data and it has not been reported any analysis showing that FDR incorrectly quantifies the rate of false positive voxels in VLSM (8). The analysis was carried out using the VLSM package version 2.60 (https://aphasialab.org/vlsm/) on MATLAB R2017a (Mathworks, Natick, MA) software. Identification of the brain regions associated with the significant voxels was made using the AAL atlas and Natbrainlab atlas of WM pathways (9) in MRICronGL (https://www.nitrc.org/projects/mricrogl) on an MNI standard brain.

2.4.3 Behavioral data analysis

We checked normality of data using the Shapiro-Wilk test and homogeneity of variance using Levene's test and conducted parametric (analysis of variance [ANOVA/ANCOVA] and independent t-tests) or non-parametric (Kruskal-Wallis and Mann-Whitney U tests) statistical tests as appropriate. If needed, post hoc tests were conducted (P< 0.05, two-tailed) with Bonferroni correction for multiple comparisons. Effect sizes [Eta square: η^2^=0.01 indicates a small effect size, η^2^=0.06 a medium effect size and η^2^=0.14 a large effect size, and Cohen’s d: d=0.2 indicates a small effect size, d=0.5 a medium effect size and d=0.8 a large effect size) were calculated when appropriate.

2.4.4 White matter tracts disconnection analysis

We analyzed a total of 10 tracts: association (arcuate, superior longitudinal, inferior longitudinal, cingulum, fronto-striatal, frontal superior longitudinal), commissural (corpus callosum, anterior commissure) and projection (thalamic, pons) tracts. These tracts were chosen given the cerebello-cortical connectivity network knowledge available to date (10) . For each individual participant (participants with bilateral lesions were excluded), we considered a given WM tract to be disconnected if the patient’s lesion overlapped a voxel within the WM pathway map with a probability higher than 50% (above the chance level). We then conducted chi square tests to compare the number of participants with a tract disconnection for each tract between participants with versus without a deficit in ToM as assessed by the Happe’s Strange stories and the Faux Pas Stories test. Patients were classified into groups using zero as a cut-off score, with scores lower than zero reflecting a ToM deficit. This analysis was subjected to Bonferroni correction for multiple comparisons (α level; *p* = 0.005 based on 10 tracts analyzed).

References:

1. Happé F, Brownell H, Winner E. Acquired “theory of mind” impairments following stroke. *Cognition* (1999) 70:211–240. doi:10.1016/S0010-0277(99)00005-0

2. Fletcher PC, Happe F, Frith U, Baker SC, Dolan RJ, Frackowiak RSJ, Frith CD. Other minds in the brain: a functional imaging study of “theory of mind” in story comprehension. *Cognition* (1995) 57:109–128.

3. White S, Hill E, Happé F, Frith U. Revisiting the strange stories: Revealing mentalizing impairments in autism. *Child Dev* (2009) 80:1097–1117. doi:10.1111/j.1467-8624.2009.01319.x

4. Solomon J, Raymont V, Braun A, Butman JA, Grafman J. User-friendly software for the analysis of brain lesions (ABLe). *Comput Methods Programs Biomed* (2007) 86:245–254.

5. Tzourio-Mazoyer N, Landeau B, Papathanassiou D, Crivello F, Etard O, Delcroix N, Mazoyer B, Joliot M. Automated anatomical labeling of activations in SPM using a macroscopic anatomical parcellation of the MNI MRI single-subject brain. *Neuroimage* (2002) 15:273–289.

6. Collins DL, Neelin P, Peters TM, Evans AC. Automatic 3D intersubject registration of MR volumetric data in standardized Talairach space. *J Comput Assist Tomogr* (1994) 18:192–205.

7. Woods RP, Mazziotta JC, R. Cherry and S. MRI-PET Registration with Automated Algorithm. *J Comput Assist Tomogr* (1993) 17:

8. Mirman D, Landrigan J-F, Kokolis S, Verillo S, Ferrara C, Pustina D. Corrections for multiple comparisons in voxel-based lesion-symptom mapping. *Neuropsychologia* (2018) 115:112–123. doi:10.1016/j.neuropsychologia.2017.08.025

9. Thiebaut de Schotten M, Bizzi A, Dell’Acqua F, Allin M, Walshe M, Murray R, Williams SC, Murphy DGM, Catani M. Atlasing location, asymmetry and inter-subject variability of white matter tracts in the human brain with MR diffusion tractography. *Neuroimage* (2011) 54:49–59.

10. Buckner RL, Krienen FM, Castellanos A, Diaz JC, Yeo BTT. The organization of the human cerebellum estimated by intrinsic functional connectivity. *J Neurophysiol* (2011) 106:2322–2345. doi:10.1152/jn.00339.2011
